# Impact of frontline treatment approach on outcomes in patients with secondary AML with prior hypomethylating agent exposure

**DOI:** 10.1186/s13045-022-01229-z

**Published:** 2022-01-29

**Authors:** Nicholas J. Short, Sangeetha Venugopal, Wei Qiao, Tapan M. Kadia, Farhad Ravandi, Walid Macaron, Courtney D. Dinardo, Naval Daver, Marina Konopleva, Gautam Borthakur, Elizabeth J. Shpall, Uday Popat, Richard E. Champlin, Rohtesh Mehta, Gheath Al-Atrash, Betul Oran, Elias Jabbour, Guillermo Garcia-Manero, Ghayas C. Issa, Guillermo Montalban-Bravo, Musa Yilmaz, Abhishek Maiti, Hagop Kantarjian

**Affiliations:** 1 Department of Leukemia, Unit 428, The University of Texas MD Anderson Cancer Center, 1515 Holcombe Boulevard, Houston, TX 77030 USA; 2 Department of Biostatistics, The University of Texas MD Anderson Cancer Center, Houston, TX USA; 3 Department of Stem Cell Transplantation and Cellular Therapy, The University of Texas MD Anderson Cancer Center, Houston, TX USA

**Keywords:** Acute myeloid leukemia, Azacitidine, Decitabine, Myelodysplastic syndrome, Chronic myelomonocytic leukemia

## Abstract

**Background:**

Treated secondary acute myeloid leukemia (ts-AML)—i.e., AML arising from a previously treated antecedent hematologic disorder—is associated with very poor outcomes. The optimal frontline treatment regimen for these patients is uncertain.

**Methods:**

We retrospectively analyzed 562 patients who developed AML from preceding myelodysplastic syndrome or chronic myelomonocytic leukemia for which they had received a hypomethylating agent (HMA). Patients with ts-AML were stratified by frontline AML treatment with intensive chemotherapy (IC, n = 271), low-intensity therapy (LIT) without venetoclax (n = 237), or HMA plus venetoclax (n = 54).

**Results:**

Compared with IC or LIT without venetoclax, HMA plus venetoclax resulted in higher CR/CRi rates (39% and 25%, respectively; *P* = 0.02) and superior OS (1-year OS 34% and 17%, respectively; *P* = 0.05). The benefit of HMA plus venetoclax was restricted to patients with non-adverse risk karyotype, where HMA plus venetoclax resulted in a median OS of 13.7 months and 1-year OS rate of 54%; in contrast, for patients with adverse risk karyotype, OS was similarly dismal regardless of treatment approach (median OS 3–5 months). A propensity score analysis accounting for relevant clinical variables confirmed the significant OS benefit of HMA plus venetoclax, as compared with other frontline treatment approaches. In a landmark analysis, patients with ts-AML who underwent subsequent hematopoietic stem cell transplantation (HSCT) had superior 3-year OS compared to non-transplanted patients (33% vs. 8%, respectively; *P* = 0.003).

**Conclusions:**

The outcomes of ts-AML are poor but may be improved with use of an HMA plus venetoclax-based regimen, followed by HSCT, particularly in those with a non-adverse risk karyotype.

**Supplementary Information:**

The online version contains supplementary material available at 10.1186/s13045-022-01229-z.

## Introduction

Secondary acute myeloid leukemia (s-AML) is an adverse-risk subtype of AML that is broadly comprised of AML arising from an antecedent hematologic disorder or AML that developed after exposure to cytotoxic chemotherapy or irradiation (i.e., therapy-related AML) [1]. The poor outcomes observed in s-AML are multifactorial and are driven both by the relatively older age of these patients and by higher rates of adverse-risk cytogenetics and mutations as compared with de novo AML [2–4]. Within the subgroup of patients with s-AML arising from a previously diagnosed myeloid malignancy (e.g., myelodysplastic syndromes [MDS], chronic myelomonocytic leukemia [CMML], myeloproliferative neoplasm, etc.), a significant proportion have received prior treatment with a hypomethylating agent (HMA) at the time of AML transformation. HMAs such as azacitidine and decitabine are widely used for the treatment of higher-risk MDS or CMML; however, a significant proportion of patients eventually progress to AML. These patients with AML arising from a previously treated hematologic disorder (henceforth referred to as “treated secondary AML” [ts-AML]) have a particularly poor prognosis [5, 6]. In one analysis, the median overall survival (OS) of patients with ts-AML was only 4.2 months, and the outcomes of these patients were inferior to other poor-risk subgroups, including untreated s-AML and *TP53*-mutated AML [5].

There is no clear consensus for the optimal treatment of patients with ts-AML [7]. In a randomized phase III study in patients with newly diagnosed s-AML, CPX-351—a liposomal formulation of cytarabine and daunorubicin—improved response rates and OS compared with standard 7 + 3 chemotherapy [8]. However, in a subgroup analysis of 133 patients with prior HMA exposure for an antecedent hematologic malignancy, there was no difference in outcomes between CPX-351 and 7 + 3 chemotherapy (median OS: 5.7 months vs. 5.9 months, respectively). Given the relatively older age of patients with ts-AML, these patients are often not suitable candidates for intensive chemotherapy (IC). While the combination of a HMA plus venetoclax has emerged as a new standard of care for older patients with newly diagnosed AML who are unfit for intensive therapy, patients with ts-AML were excluded from the pivotal study that led to approval of this regimen [9]. In the randomized phase III study of low-dose cytarabine with or without venetoclax for older adults with newly diagnosed AML—which did not exclude patients with prior HMA exposure—response rates and OS were significantly worse in patients with ts-AML, highlighting the adverse prognosis of these patients [10]. In light of the lack of robust data to guide treatment selection for this poor-risk AML subgroup, we performed a retrospective analysis of patients with ts-AML and evaluated the impact of different frontline therapies on clinical outcomes.

## Methods

### Study design and participants

This is a retrospective study evaluating the prognostic impact of frontline AML therapy on clinical outcomes in adults with newly diagnosed AML arising from either MDS or CMML and who had received HMA-based therapy for their preceding hematologic disorder. Patients were eligible regardless of the number of prior lines of therapy for MDS/CMML as long as at least one of these treatments consisted of an HMA-based regimen; however, patients who had received IC and/or venetoclax for MDS/CMML prior to AML diagnosis were excluded. Frontline AML treatments were divided into three categories for analysis: (1) IC without venetoclax, (2) low-intensity therapy (LIT) without venetoclax, and (3) HMA plus venetoclax, with or without a third agent. Adverse risk karyotype was defined according to European LeukemiaNet (ELN) consensus guidelines [11]. This study was conducted at a single academic center (The University of Texas MD Anderson Cancer Center [UTMDACC]). This study was approved by the Institutional Review Board of UTMDACC and was conducted in accordance with the Declaration of Helsinki.

### Mutation profiling

Mutation analysis was performed on bone marrow specimens using a 28-, 53- or 81-gene targeted NGS panel as previously described [12, 13]. Genomic DNA was extracted from bone marrow aspirates. A minimum sequencing coverage of 250X (bi-directional true paired-end sequencing) and minimum input of 250 ng of DNA were required. The analytical sensitivity was established at 5% mutant reads in a background of wild-type reads. Established bioinformatics pipelines were used to identify somatic variants.

### Response and outcome definitions

Complete remission (CR), CR with incomplete hematologic recovery (CRi), and morphologic leukemia-free state (MLFS) were defined according to ELN consensus guidelines [11]. Relapse was defined as recurrence of bone marrow blasts > 5% or extramedullary AML. Cumulative incidence of relapse (CIR) was calculated from the time of CR/CRi/MLFS until relapse, censored for death in morphological remission or if the patient was alive at last follow-up. Relapse-free survival (RFS) was calculated from the time of CR/CRi/MLFS until relapse or death from any cause, censored if the patient was alive at last follow-up. OS was calculated from the time of treatment initiation until death from any cause, censored if the patient was alive at last follow-up. Survival estimates were not censored at the time of allogeneic hematopoietic stem cell transplantation (HSCT).

### Statistical methods

Patient characteristics were summarized using median (range) for continuous variables and frequencies (percentages) for categorical variables. To compare two groups, Fisher’s exact test was performed for categorical variables, and Wilcoxon rank-sum test was performed for continuous variables. The Kaplan–Meier method was used to estimate the probabilities for RFS and OS, and differences between groups were evaluated with the log-rank test. The Gray’s test was used to compare cumulative incidence probabilities between groups. For the propensity score analysis, generalized boosted models (GBM) were used to estimate the propensity score weights. The GBM is a machine-learning technique which involves an iterative process with multiple regression tree able to capture the complex relationship between treatment assignments and pretreatment covariates without overfitting [14]. R package “twang” was used to obtain propensity score weight based on GBM. All patients are included in the resultant propensity score analysis. Statistical analyses related to the propensity score analysis were carried out in SAS version 9.4 and R version 4.1.1. All other statistical analyses were performed using GraphPad Prism 8.

## Results

### Patient characteristics and study cohort

Between June 2004 and January 2021, we identified 562 patients with ts-AML who received frontline AML therapy at our institution. Overall, 271 patients (48%) received IC as frontline AML therapy, 237 (42%) received LIT without venetoclax, and 54 (10%) received HMA plus venetoclax. The regimens received are shown in Additional file 1: Table S1. Baseline characteristics of the study population are shown in Table 1. The median age of the overall population was 69 years (range 21–92 years). As expected, patients treated with IC were generally younger (median age: 65 years for IC group vs. 73 years for patients treated with other regimens; *P* < 0.0001). The median number of prior therapies for antecedent hematologic disorder was 1 (range 1–5), and 41% had received ≥ 2 prior therapies prior to AML diagnosis. Overall, 10% had undergone prior allogeneic HSCT for MDS or CMML. The population was enriched with poor-risk mutations, including an *ASXL1* mutation in 28%, *RUNX1* mutation in 18%, and *TP53* mutation in 27%. Among the 54 patients who received HMA plus venetoclax as first AML therapy, 31 (57%) switched HMA at the time of progression to AML and 23 (43%) continued the same HMA. Twenty-four patients (45% of the HMA plus venetoclax cohort) also received a third agent as part of their frontline AML regimen, including a FLT3 inhibitor in 4 patients, an IDH1 or IDH2 inhibitor in 4 patients, gemtuzumab ozogamicin in 2 patients, and other investigational agents in 14 patients.

**Table 1:** Baseline characteristics of the study population (*N* = 562)

Characteristic^a^	IC (*N* = 271)	LIT without Ven (*N* = 237)	HMA + Ven (*N* = 54)	*P* value
*Age*				
Median (years)	65 (21–91)	73 (53–92)	71 (42–84)	< 0.0001
≥ 60 years	191 (70)	226 (95)	50 (93)	< 0.0001
*Preceding diagnosis*				
MDS	236 (87)	204 (86)	43 (80)	0.35
CMML	35 (13)	33 (14)	11 (20)	
*Prior therapies*				
Prior allogeneic HSCT	31 (11)	13 (5)	10 (19)	0.005
Median number of prior therapies	1 (1–5)	1 (1–5)	1 (1–3)	0.65
*Cytogenetics*				
Diploid	82 (30)	91 (38)	16 (30)	0.31
Non-diploid, non-adverse	56 (21)	45 (19)	7 (13)	
Adverse	111 (41)	81 (34)	25 (46)	
IM/ND	22 (8)	20 (9)	6 (11)	
*Mutation*				
*ASXL1*	36/102 (35)	24/101 (26)	13/54 (24)	0.14
*DNMT3A*	15/139 (11)	23/150 (15)	12/54 (22)	0.12
*FLT3 D835*	3/247 (1)	7/232 (3)	0	0.38
*FLT3-*ITD	12/247 (5)	25/232 (11)	1/54 (2)	0.01
*IDH1*	10/166 (6)	10/171 (6)	6/54 (11)	0.37
*IDH2*	12/167 (7)	11/170 (6)	4/54 (7)	0.94
*NPM1*	11/191 (6)	14/189 (7)	1/54 (2)	0.31
*KRAS/NRAS*	37/241 (15)	47/224 (21)	7/54 (13)	0.18
*RUNX1*	23/101 (23)	26/101 (26)	16/54 (30)	0.64
*TET2*	25/107 (23)	27/104 (26)	12/54 (22)	0.85
*TP53*	46/144 (32)	31/152 (20)	18/54 (33)	0.04

^a^Continuous variables are listed as median [range] and categorical variables as n (%) or n/N (%)

*IC* intensive chemotherapy, *LIT* low-intensity therapy, *Ven* venetoclax, *HMA* hypomethylating agent, *MDS* myelodysplastic syndrome, *CMML* chronic myelomonocytic leukemia, *HSCT* hematopoietic stem cell transplantation, *IM/ND* insufficient metaphases/not done

### Outcomes of the global study population

For the entire study population, the CR rate was 16%, the CR/CRi rate was 26%, and the CR/CRi/MLFS rate was 35%. The 30-day and 60-day mortality rates were 9% and 14%, respectively. The median duration of follow-up for the entire cohort was 47 months. The median duration of response was 7.7 months, with 1-year and 2-year CIR rates of 63% and 75%, respectively. Median RFS was 5.7 months, with 1-year and 2-year RFS rates of 26% and 17%, respectively. Median OS was 4.8 months, with 1-year and 2-year OS rates of 19% and 7%, respectively.

### Response rates and early mortality by treatment approach

Responses by treatment approach are shown in Table 2. Response rates were similar between patients who received IC or LIT without venetoclax. In contrast, response rates were superior for patients who received HMA plus venetoclax. Treatment with HMA plus venetoclax resulted in higher CR/CRi rates compared with IC or LIT without venetoclax (39% vs. 25%, respectively; *P* = 0.02). Similarly, rates of CR/CRi/MLFS were higher with HMA plus venetoclax as compared with IC or LIT without venetoclax (54% vs. 32%, respectively; *P* = 0.002). The increased rates of response with HMA plus venetoclax were primarily observed in patients with non-adverse risk karyotype. Among those with non-adverse karyotype, the CR/CRi rates with HMA plus venetoclax versus IC/LIT without venetoclax were 57% and 30%, respectively (*P* = 0.008). However, CR/CRi rates were low in patients with ts-AML and adverse karyotype, regardless of treatment approach (18% and 16%, respectively; *P* = 0.8).

**Table 2:** Response rates by treatment approach

Response^a^	IC (*N* = 271)	LIT without Ven (*N* = 237)	HMA + Ven (*N* = 54)	*P* value (LIT without Ven vs. HMA + Ven)	*P* value (IC vs. HMA + Ven)
CR	43 (16)	34 (14)	14 (26)	0.03	0.08
CRi	21 (7)	27 (11)	7 (13)	0.68	0.14
MLFS	18 (6)	22 (9)	8 (15)	0.19	0.02
*CR/CRi*	64 (24)	61 (26)	21 (39)	0.06	0.02
*CR/CRi/MLFS*	82 (30)	83 (35)	29 (54)	0.01	< 0.001
No response	152 (56)	112 (47)	18 (33)	0.06	0.002
Early death/not evaluable	37 (14)	42 (18)	7 (13)	0.38	0.84

^a^Responses are shown as n (%)

*IC* intensive chemotherapy, *LIT* low-intensity therapy, *Ven* venetoclax, *CR* complete remission, *CRi* CR with incomplete hematologic recovery, *MLFS* morphologic leukemia-free state

Early mortality rates were similar among the 3 treatment groups. The 30-day mortality rates of IC, LIC without venetoclax and HMA plus venetoclax were 10%, 9% and 7%, respectively (*P* = 0.74), and the 60-day mortality rates were 25%, 22% and 20%, respectively (*P* = 0.71).

### Survival outcomes by treatment approach

**Fig. 1:**
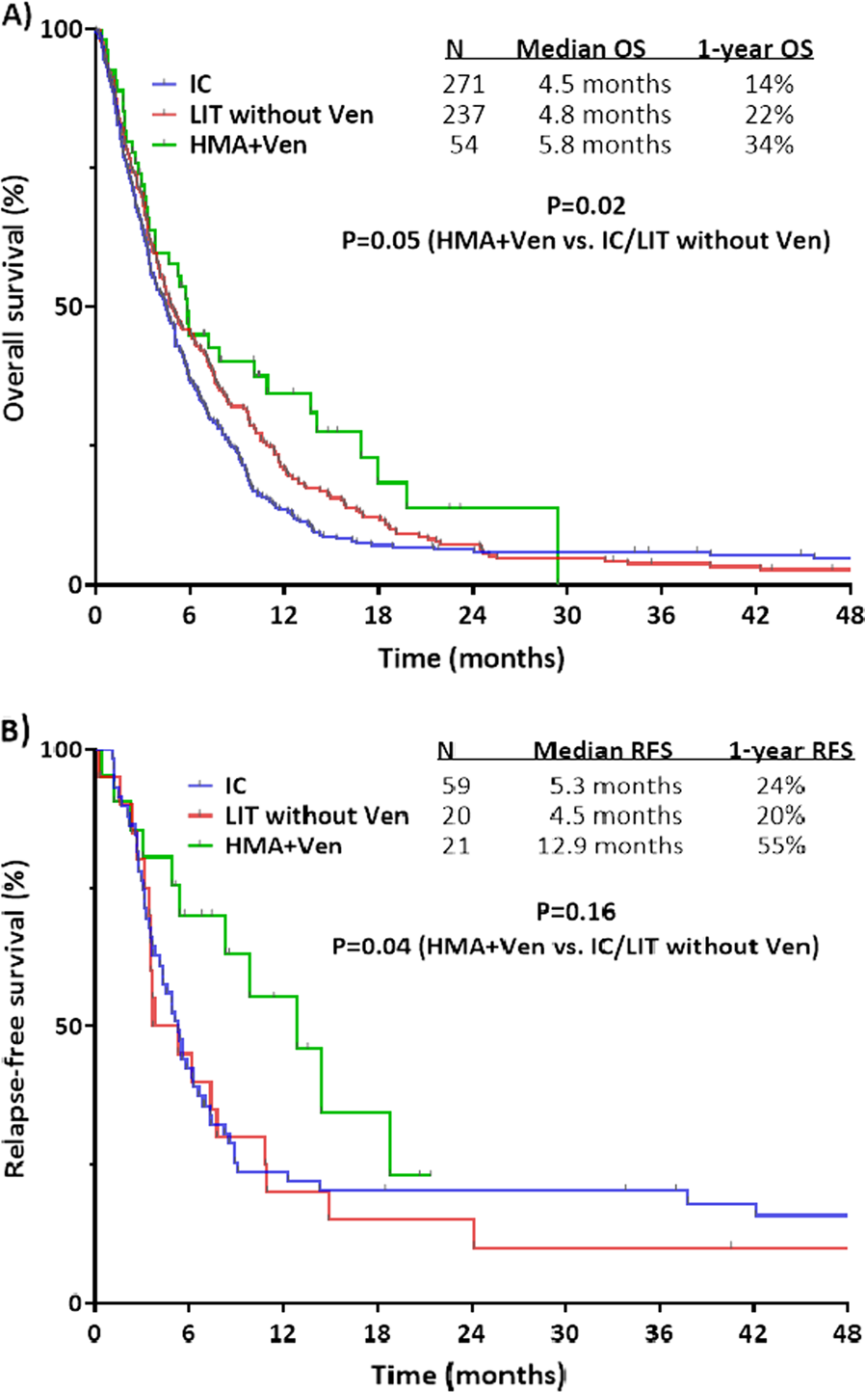
Outcomes of treated secondary AML by treatment approach. (**A**) overall survival and (**B**) relapse-free survival

Among patients treated with HMA plus venetoclax, the median OS was 5.8 months and the 1-year OS rate was 34%, which was superior to outcomes with IC (median OS of 4.5 months and 1-year OS rate of 14%) or LIT without venetoclax (median OS of 4.8 months and 1-year OS rate of 22%) (Fig. 1A). The outcomes of patients who received HMA plus venetoclax were significant superior to the combined group of patients who received IC or LIT without venetoclax, with a 1-year OS rate that was double that of the IC/LIT without venetoclax group (34% vs. 17%, respectively; *P* = 0.05). RFS was also superior in patients who received HMA plus venetoclax as compared with IC/LIT without venetoclax (median RFS of 12.9 months vs. 5.3 months and 1-year OS rates of 55% and 22%, respectively; *P* = 0.04; Fig. 1B).

OS was similar among patients who received an HMA plus venetoclax alone and those who received a triplet regimen of an HMA, venetoclax, and a third agent (*P* = 0.99), suggesting that the addition of a third agent did not confound our findings of the superiority of an HMA plus venetoclax-based regimen in ts-AML. Furthermore, when patients were stratified by treatment for ts-AML before or after 11/2017—which was the beginning of the widespread use of venetoclax on clinical trials at our institution—there was no impact of treatment era on outcomes. OS pre-11/2017 and post-11/2017 was similar within both the IC without venetoclax cohort (*P* = 0.48) and the LIT without venetoclax cohort (*P* = 0.28). Thus, the fact that patients who received HMA plus venetoclax were more likely to be treated in the contemporary era also did not appear to impact our findings. Notably, among patients treated with an HMA plus venetoclax, there was also no difference in outcomes between patients who switched HMA at the time of AML progression and those who continued with the same HMA (*P* = 0.82).

**Fig. 2:**
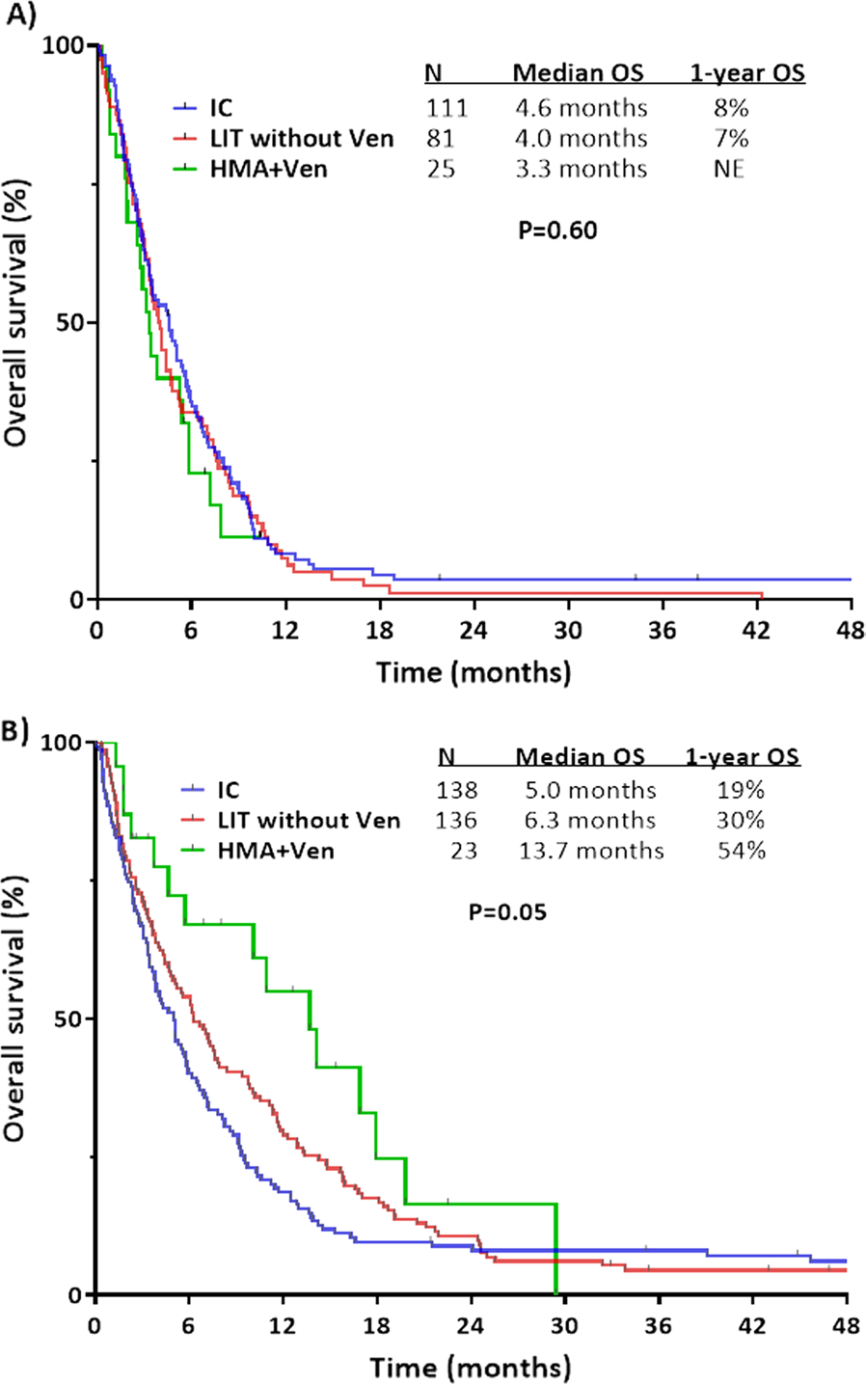
Overall survival of treated secondary AML by treatment approach and karyotype risk. (**A**) Adverse risk karyotype and (**B**) non-adverse risk karyotype

Given the established impact of age and karyotype on outcomes in AML, we performed additional subgroups analyses to clarify the impact of treatment approach, accounting for these relevant variables. Among patients with adverse risk karyotype at the time of AML transformation, outcomes were similarly poor regardless of treatment approach. The median OS for patients treated with IC, LIT without venetoclax or HMA plus venetoclax was 4.6 months, 4.0 months and 3.3 months, respectively (*P* = 0.60) (Fig. 2A). Conversely, among patients with non-adverse risk karyotypes, OS was superior in patients who received HMA plus venetoclax, a group in whom a median OS of 13.7 months and a 1-year OS rate of 54% were observed (Fig. 2B). The outcomes with HMA plus venetoclax in this non-adverse risk cytogenetic group was statistically superior to those who received IC (median OS of 5.0 months and 1-year OS rate of 19%; *P* = 0.01) and was numerically superior to those who received LIT without venetoclax (median OS of 6.3 months and 1-year OS rate of 30%; *P* = 0.12). Compared to the combined group of patients who received IC/LIT without venetoclax, HMA plus venetoclax resulted in significant improved OS, with more than double the median OS and 1-year OS rates (median OS 13.7 months vs. 5.5 months; 1-year OS rates 55% vs. 24%, respectively; *P* = 0.04).

The benefit of HMA plus venetoclax was observed in most molecular subgroups, with the exception of *TP53*-mutated ts-AML (Additional file 1: Fig. S1). In patients with *NPM1*, *IDH1* and/or *IDH2* mutation(s), all of which have previously been shown to be relatively sensitive to venetoclax-based regimens [15, 16], HMA plus venetoclax was associated superior OS as compared with IC or LIT without venetoclax (median OS 16.9 months vs. 5.5 months; 1-year OS rates 60% vs. 26%; *P* = 0.06). Similarly, HMA plus venetoclax was associated with superior OS in patients with *ASXL1* mutations (median OS 17.9 months vs. 6.3 months; 1-year OS rate 66% vs. 22%; *P* = 0.01) or with *RUNX1* mutations (median OS 14.1 months vs. 7.3 months; 1-year OS rate 65% vs. 23%; *P* = 0.005). Conversely, among patients with *TP53*-mutated ts-AML, outcomes were inferior with HMA plus venetoclax as compared with IC/LIT without venetoclax (median OS 2.8 months vs. 3.5 months; 1-year OS rate 3% vs. 0%; *P* = 0.06).

**Fig. 3:**
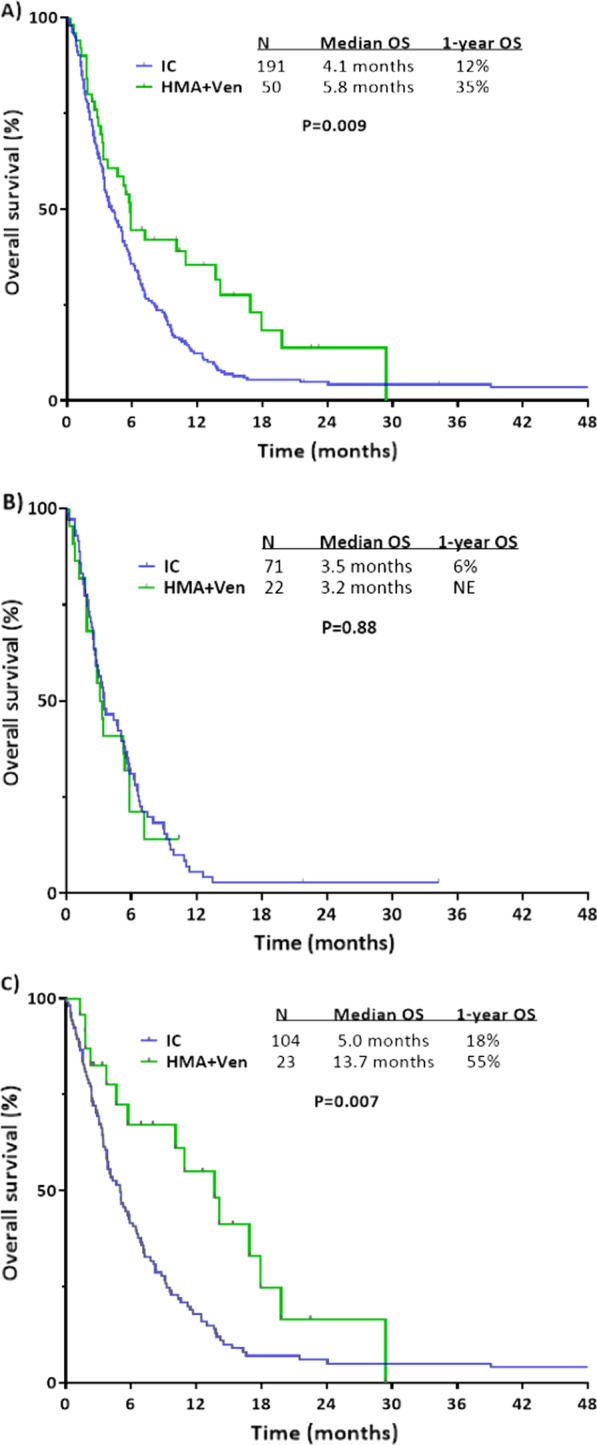
Overall survival of patients ≥ 60 years of age with treated secondary AML by treatment approach and karyotype risk. (**A**) All patients, (**B**) adverse risk karyotype only, and (**C**) non-adverse risk karyotype only

Given the discrepancy in age between patients treated with IC (30% of whom were < 60 years of age) and those treated with HMA plus venetoclax (7% of whom were < 60 years of age), we performed additional analyses limited to patients ≥ 60 years of age. Among these older patients, the OS benefit associated with HMA plus venetoclax compared with IC was particularly pronounced (median OS of 5.8 months vs. 4.1 months and 1-year OR rates of 35% vs. 12%, respectively; *P* = 0.009; Fig. 3A). Among patients with adverse risk karyotypes, outcomes were again similar between these 2 treatment approaches (median OS 3.5 months for IC vs. 3.2 months for HMA plus venetoclax; *P* = 0.88; Fig. 3B), and the superior OS associated with HMA plus venetoclax was restricted to those patients with non-adverse risk karyotype (median OS of 13.7 months vs. 5.0 months and 1-year OR rates of 55% vs. 18%, respectively; *P* = 0.007; Fig. 3C). Due to increased risk of treatment-related complications and mortality, IC may not be optimal for many patients ≥ 60 years of age [17], and thus we also compared outcomes in patients age < 60 years who received IC to those of any age who received HMA plus venetoclax (93% of whom were ≥ 60 years of age). Despite the older age of the HMA plus venetoclax group, their outcomes were numerically superior to the younger IC group (median OS of 5.8 months vs. 5.0 months and 1-year OS rates of 34% vs. 17%, respectively; *P* = 0.16; Additional file 1: Fig. S2).

### Propensity score analysis comparing treatment approaches

To account for differences in baseline variables among the 3 groups, we performed a propensity score analysis. The propensity score weightings were based on age, prior MDS versus CMML, number of prior therapies for antecedent hematologic disorder, karyotype, prior allogeneic HSCT, and presence of *ASXL1*, *RUNX1* or *TP53* mutations. After propensity score weighting, the covariates between treatments were balanced, showing no significant difference across arms (Additional file 1: Table S2). In this analysis after using the propensity score to control for imbalances of the pretreatment variables, HMA plus venetoclax was associated with higher CR/CRi/MLFS rates compared to either IC (odds ratio [OR] 3.09 [95% CI 1.62–5.88]; *P* < 0.001) or LIT without venetoclax (OR 3.04 [95% CI 1.58–5.85]; *P* < 0.001). A similar benefit was seen with respect to CR/CRi rates (HMA plus venetoclax vs. IC: OR 2.39 [95% CI 1.23–4.65]; *P* = 0.01; HMA plus venetoclax vs. LIT without venetoclax: OR 2.66 [95% CI 1.36–5.21]; *P* = 0.004). HMA plus venetoclax was also associated with superior OS compared to both IC (hazard ratio [HR] 0.56 [95% CI 0.37–0.84; *P* = 0.005) and LIT without venetoclax (HR 0.61 [95% CI 0.41–0.92]; *P* = 0.02).

### Impact of HSCT on survival outcomes

Fifty-one patients (9% of the entire study population and 32% of patients who achieved CR/CRi/MLFS) underwent allogeneic HSCT in first AML remission. Among patients ≤ 65 years of age (n = 193), 15% underwent subsequent HSCT, which included 52% of responders in this age group. Among the 51 transplanted patients, the median time from start of therapy to HSCT was 2.6 months (range 1.3–7.9 months). Overall, 34 patients underwent HSCT after IC, 12 after LIT without venetoclax and 5 after HMA plus venetoclax. The rate of subsequent HSCT was higher in those who had received IC as first AML therapy (41% of responders) versus those who received LIT without venetoclax (14% of responders) or HMA plus venetoclax (17% of responders). Five patients who underwent HSCT in first remission for AML had also undergo prior allogeneic HSCT for preceding MDS or CMML, including 4 patients in the IC group and 1 patient in the LIT without venetoclax group.

**Fig. 4:**
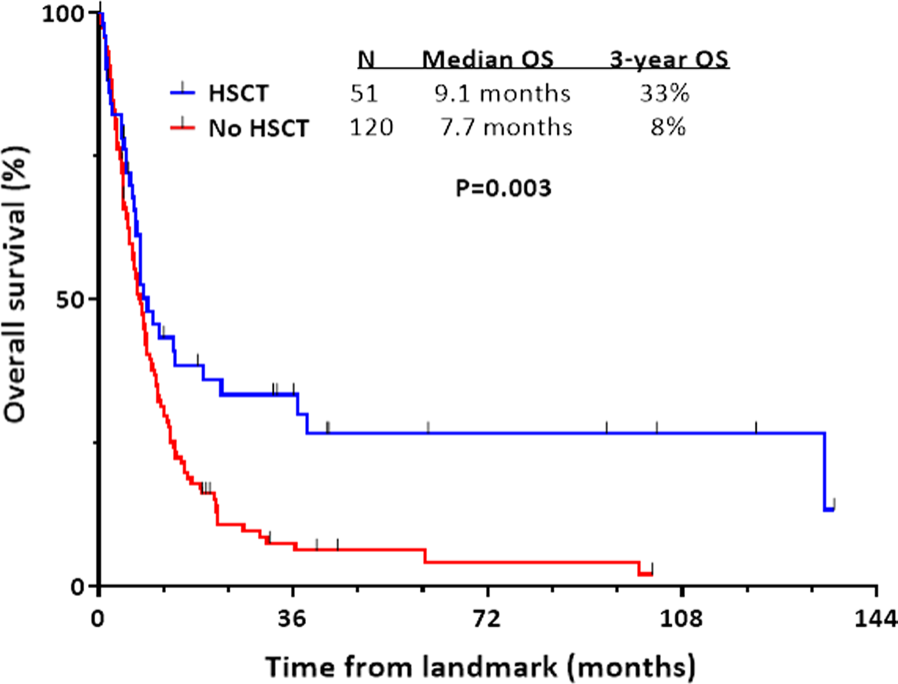
Overall survival landmark analysis of patients with treated secondary AML who underwent HSCT in first remission versus those who did not undergo transplant. Landmark analysis was performed from time of HSCT in the HSCT group and from the landmark time point (2.6 months from start of AML therapy) in the non-HSCT group

Among the 143 responding patients who did not undergo HSCT, 120 patients were included in a landmark analysis and 23 patients were excluded who either relapsed (n = 14), died in remission (n = 7) or were lost to follow-up (n = 2) within 2.6 months of AML diagnosis (i.e., the median time to HSCT). HSCT in first remission was associated with a significant improvement in OS compared to no HSCT (median OS 9.1 months and 3-year OS 33% vs. median OS 7.7 months and 3-year OS 8%, respectively; *P* = 0.003; Fig. 4). Among the 51 transplanted patients, 15 (29%) were still alive without relapse at last follow-up, 2 (4%) relapsed but were still alive at last follow-up, 19 (37%) had relapsed and died, and 15 (29%) had died in remission. Among the 15 deaths in remission, 12 were considered to be HSCT-related (5 from graft vs. host disease and 7 from other causes). Outcomes of patients who received IC followed by HSCT were similar to those who received LIT without venetoclax followed by HSCT (3-year OS rates: 33% and 25%, respectively; Additional file 1: Fig. S3 ). Among the 5 patients who received HMA pus venetoclax followed by HSCT, all were still alive at last follow-up with a median post-HSCT duration of follow-up of 10.3 months (range 8.0–14.8 months).

## Discussion

In this study, we have shown that response rates and survival of patients with ts-AML are superior when an HMA plus venetoclax-based regimen—rather than either IC or LIT without venetoclax—is used as frontline AML therapy. These findings were confirmed with a propensity score weighted analysis to control for imbalances among relevant baseline variables. The beneficial impact of HMA plus venetoclax as a frontline treatment approach for ts-AML appeared to be restricted to those patients with a non-adverse karyotype, while outcomes were similarly poor in patients with ts-AML and an adverse karyotype, regardless of treatment approach. Outcomes were further improved when patients with ts-AML underwent subsequent HSCT, where a 3-year post-HSCT OS rate of 33% was observed. Together, these findings suggest that frontline treatment with an HMA plus venetoclax-based regimen, followed by allogeneic HSCT for fit patients, may be the optimal treatment strategy for patients with ts-AML.

Our study confirms the very poor outcomes of patients with ts-AML. In the global population, the median OS was only 4.8 months, and the 2-year OS rate was < 10%. However, both response rates and OS were significantly better in patients without an adverse karyotype, particularly when an HMA plus venetoclax was used as frontline therapy. In this group of patients with non-adverse karyotype ts-AML treated with HMA plus venetoclax, the median OS was 13.7 months, with a 1-year OS rate of 54%. Acknowledging the relatively small number of HMA plus venetoclax-treated patients in our analysis, these results compare relatively favorably to the outcomes reported in a randomized study with where the median OS was 14.7 months with azacitidine plus venetoclax in a population of older and unfit patients, none of whom had ts-AML [9]. While the long-term outcomes with HMA plus venetoclax in ts-AML remain suboptimal for these patients with non-adverse karyotype, our findings support the strong consideration of an HMA plus venetoclax-based regimen in this population, rather than either IC or LIT without venetoclax. Recent studies have reported very promising outcomes with IC plus venetoclax for patients with newly diagnosed AML, with response rates of 90–95% and 1-year OS rates of approximately 95% [18, 19]. As our analysis did not include patients treated with IC plus venetoclax, it remains possible that this approach might be superior to HMA plus venetoclax in patients with non-adverse karyotype ts-AML. However, given the relatively older age of patients with ts-AML (median age 69 years of age in our cohort), careful selection of these patients will be imperative.

Despite the superior outcomes observed with HMA plus venetoclax in patients with non-adverse karyotype, we observed similarly dismal outcomes for patients with adverse karyotype ts-AML, regardless of the chosen frontline AML therapy. These patients with adverse karyotype ts-AML represented approximately 40% of all patients with ts-AML, and their median OS was < 5 months and 1-year OS was < 10% across treatment approaches. The extremely poor outcome of this subgroup of patients has several important implications. First, it should be noted that the survival outcomes of these patients are much more akin to relapsed/refractory AML than to newly diagnosed AML. Historically, a 1-year OS of approximately 30% has been reported for patients with AML in first relapse [20], and preliminary data suggest that these outcomes may further improve with the use of venetoclax-based regimens in the relapsed/refractory setting [18, 19, 21]. Despite ts-AML sometimes being classified as “newly diagnosed” AML, it may be more appropriate to enroll patients with ts-AML (particularly those with adverse karyotype) into clinical trials designed for patients with relapsed/refractory disease. Furthermore, given the established chemoresistance of this AML subtype and the lack of any known effective treatments, phase I clinical trials with novel agents may be appropriate for these patients, even in the frontline setting.

Several studies have reported the impact of HSCT in patients with s-AML [22–24]; however, there is a paucity of data supporting the use of consolidative HSCT in patients specifically with ts-AML. Our analysis suggests that allogeneic HSCT is a potential curative strategy in patients with ts-AML and also highlights the dismal outcome of patients who are unable to proceed to HSCT. We observed that patients who underwent HSCT in first remission had a 3-year OS rate of 33%, whereas the 3-year OS rate was only 8% in non-transplanted patients. These results suggest that proceeding to HSCT should be a primary goal for patients with ts-AML. However, the advanced age of patients with ts-AML, combined with their relatively low rate of response to conventional therapies, makes it challenging to bridge many of these patients to HSCT. In our cohort, only 9% of patients overall and 32% of all responders underwent HSCT in first remission. While a limitation of our analysis is that we do not have clear documentation of why many responders did not proceed to HSCT, in many cases this decision was presumably driven by the patient’s perceived lack of fitness of HSCT. Thus, in addition to novel regimens capable of improving response rates in ts-AML, the development of safer and more effective transplant regimens for older adults with AML is imperative to bridging more of these patients to potentially curative HSCT.

This study has several limitations. This is a retrospective analysis performed at a large academic center, with many patients treated on clinical trials. The outcomes observed with these regimens may therefore not be reflective of those in community or smaller academic practices. Additionally, it was necessary to group patients according to broad therapeutic approaches for our analyses (i.e., IC or LIT without venetoclax and HMA plus venetoclax), although some heterogeneity remained within these groups. For example, nearly half of the patients in the HMA plus venetoclax group received a third agent as part of a clinical trial (or other off-label use). However, we did not observe any impact of this additional therapy on clinical outcomes. The number of patients with ts-AML who were treated with an HMA plus venetoclax was also relatively small (n = 54) as compared with other regimens, and this number was even smaller for certain subgroups analyses (e.g., the impact of karyotype and treatment approach on outcomes). Our confidence in the true response rate and survival outcomes with an HMA plus venetoclax is thus weaker than with these other therapies. However, it is notable that the rates of CR/CRi and CR/CRi/MLFS we observed with this approach (29% and 54%, respectively) are similar to the rate of CR + marrow CR observed in one prospective and one retrospective study of HMA plus venetoclax in patients with MDS after HMA failure (reported as 40% and 44%, respectively) [25, 26].

## Conclusions

In conclusion, we have shown that frontline treatment with an HMA plus venetoclax results in higher response rates and improved OS in patients with ts-AML, as compared with either IC or LIT without venetoclax, particularly in patients with a non-adverse karyotype. Outcomes of patients with ts-AML were significantly improved with HSCT in first remission, although it remains challenging to bridge many of these patients to potentially curative HSCT. While relatively favorable outcomes can be achieved with an HMA plus venetoclax-based regimen in patients with ts-AML without an adverse karyotype, outcomes are universally dismal in patients with ts-AML with an adverse karyotype, highlighting the unmet need for novel and effective strategies in this population.

## Supplementary Information

**Additional file 1.** Supplemental tables and figures.

## Data Availability

The datasets used and/or analyzed during the current study are available from the corresponding author on reasonable request.
