# Supplementary material for: Impact of frontline treatment approach on outcomes in patients with secondary AML with prior hypomethylating agent exposure
Source: J Hematol Oncol. 2022 Jan 29;15:12. doi: 10.1186/s13045-022-01229-z (PMC8800349; doi:10.1186/s13045-022-01229-z)
Supplement: Supplementary file 1 — Additional file 1. Supplemental tables and figures. [file 13045_2022_1229_MOESM1_ESM.docx]

**Title:** Impact of frontline treatment approach on outcomes in patients with secondary AML with prior hypomethylating agent exposure

Supplementary Information

**Supplementary Table 1.** Frontline treatment regimens

**Supplementary Table 2.** Standardized effect size for pretreatment covariates in the propensity score weighted and unweighted data

**Supplementary Figure 1.** Overall survival patients with treated secondary AML by treatment approach and molecular mutation(s). (A) *NPM1*, *IDH1*, and/or *IDH2*-mutated AML, (B) *ASXL1*-mutated AML, (C) *RUNX1*-mutated AML, and (D) *TP53*-mutated AML

**Supplementary Figure 2.** Overall survival of patients with treated secondary AML who received frontline intensive chemotherapy (age <60 years only) or a hypomethylating agent plus venetoclax (all ages)

**Supplementary Figure 3.** Overall survival in patients who underwent HSCT for treated secondary AML by frontline treatment approach

**Supplementary Table 1.** Frontline treatment regimens

| **Type** | **Regimen** | **N (%)** |
| --- | --- | --- |
| **Intensive Chemotherapy**  (N=271) | 7+3 ± other agent | 7 (3) |
|  | BIDFA ± other agent | 82 (30) |
|  | CIA ± other agent | 28 (10) |
|  | CLIA ± other agent | 33 (12) |
|  | CPX-351 ± other agent | 53 (20) |
|  | FIA ± other agent | 9 (3) |
|  | IA ± other agent | 42 (16) |
|  | Other high-dose cytarabine regimen | 17 (6) |
| **Low-Intensity Therapy without Venetoclax**  (N=237) | Clofarabine ± other agent | 16 (7) |
|  | Investigational agent ± HMA | 73 (31) |
|  | LDAC ± other agent | 148 (62) |
| **Hypomethylating Agent plus Venetoclax** (N=54) | HMA + venetoclax | 30 (55) |
|  | HMA + venetoclax + other agent | 24 (45) |

Abbreviations: BIDFA, twice daily fludarabine and cytarabine; CIA: clofarabine, idarubicin and cytarabine; CLIA, cladribine, idarubicin and cytarabine; FIA, fludarabine, idarubicin and cytarabine; IA, idarubicin and cytarabine; HMA, hypomethylating agent; LDAC, low-dose cytarabine

**Supplementary Table 2.** Standardized effect size for pretreatment covariates in the propensity score weighted and unweighted data

| Variable | Unweighted analysis | | Weighted analysis | |
| --- | --- | --- | --- | --- |
|  | Max standard effect size | Min p-value | Max standard effect size | Min p-value |
| Age (<60 years vs. ≥60 years) | 0.663 | <0.001 | 0.218 | 0.09 |
| MDS vs. CMML | 0.214 | 0.204 | 0.091 | 0.561 |
| Number of prior therapies | 0.167 | 0.185 | 0.266 | 0.081 |
| Cytogenetics (adverse vs. others) | 0.362 | 0.064 | 0.316 | 0.159 |
| Prior HSCT | 0.442 | 0.015 | 0.117 | 0.453 |
| *ASXL1* mutation | 0.558 | 0.07 | 0.397 | 0.543 |
| *RUNX1* mutation | 0.485 | 0.361 | 0.427 | 0.464 |
| *TP53* mutation | 0.455 | 0.023 | 0.22 | 0.713 |

**Supplementary Figure 1.** Overall survival patients with treated secondary AML by treatment approach and molecular mutation(s). (A) *NPM1*, *IDH1*, and/or *IDH2*-mutated AML, (B) *ASXL1*-mutated AML, (C) *RUNX1*-mutated AML, and (D) *TP53*-mutated AML

**Supplementary Figure 2.** Overall survival of patients with treated secondary AML who received frontline intensive chemotherapy (age <60 years only) or a hypomethylating agent plus venetoclax (all ages)

**Supplementary Figure 3.** Overall survival in patients who underwent HSCT for treated secondary AML by frontline treatment approach
